# Exploring Mental Health Literacy and Quality of Life in Multiple Sclerosis: A Cross-Sectional Study

**DOI:** 10.1097/JNN.0000000000000880

**Published:** 2026-02-25

**Authors:** Francesco Pastore, Valentina Simonetti, Barbara Forastefano, Emanuela Domenicone, Elena Amelio, Tommaso Guerra, Giancarlo Cicolini, Dania Comparcini

**Affiliations:** 1 Francesco Pastore,Tommaso Guerra and Dania Comparcini“Aldo Moro” University of Bari, Bari, Italy; 2 Valentina Simonetti and Giancarlo Cicolini, “G.d ’Annunzio” University of Chieti-Pescara, Chieti, Italy; 3 Barbara Forastefano, “TorVergata” University, Rome, Italy; 4 Emanuela Domenicone, San Filippo Neri Hospital, Asl Rome 1, Rome, Italy; 5 Elena Amelio, “Lorenzo Bonomo Hospital”, Andria, BAT, Italy

## Abstract

**BACKGROUND:** Mental health literacy (MHL) is a key factor influencing mental health outcomes and may affect quality of life (QoL) in people with multiple sclerosis (pwMS), who often experience psychiatric comorbidities. However, research on MHL in multiple sclerosis remains limited. Therefore, this study aims to investigate levels of MHL in pwMS and the relationship with their QoL and sociodemographic factors. **METHODS:** A multicenter, cross-sectional study was conducted between March and August 2024. Data were collected through an anonymous online survey consisting of a questionnaire divided into 4 sections: sociodemographic and clinical characteristics; patient experience with nursing care; the Mental Health Literacy Questionnaire-Short Version for adults (MHLq-SVa) assessing MHL across 4 domains; and the Multiple Sclerosis Quality of Life Questionnaire to assess QoL. Statistical analyses included psychometric validation procedures. **RESULTS:** A total of 170 adult pwMS participated. Participants showed moderate MHL levels, with better knowledge of mental health problems than in other domains. Higher MHL was significantly associated with female gender and higher educational attainment. Weak correlations were found between MHL and QoL, with only 1 domain: knowledge of mental health problems, which showed a significant association. The MHLq-SVa demonstrated good internal consistency (α=0.816) and construct validity. **CONCLUSION:** While MHL appears to be influenced by gender and education, its direct impact on QoL in pwMS remains limited. These findings indicate that improving MHL alone may be insufficient to enhance well-being, and that additional factors must also be addressed. Nurses are strategically positioned to assess and support MHL, and validated tools like the MHLq-SVa can guide targeted educational strategies within multidisciplinary care.

## Background

Multiple sclerosis (MS) is a chronic and unpredictable neurological disease characterized by progressive disability, cognitive decline, and a high burden of psychiatric comorbidities.^1^ Depression and anxiety are particularly prevalent and substantially increase the risk of mental health (MH) disorders among people with multiple sclerosis (pwMS),^2^ and significantly influence clinical outcomes such as treatment adherence, higher relapse rates, faster disability progression, and reduced survival.^3^ Despite the impact of MH in MS, many pwMS do not seek psychological support due to stigma, low awareness, and limited knowledge.^4^ In this context, mental health literacy (MHL) is a critical determinant. MHL encompasses the knowledge and beliefs that support the recognition, management, and prevention of mental disorders.^5^ It includes the ability to identify symptoms, understand risk factors and treatment options, use self-help strategies, seek professional support, and navigate MH information sources.^6^ Evidence shows that in MS, higher MHL is associated with better quality of life (QoL) through greater self-efficacy, adaptive coping, reduced emotional distress, and increased empowerment.^7^ It enables patients to recognize early signs of distress, enhances self-management, supports healthier behaviors, and encourages engagement in integrated care. These improvements are closely linked to better QoL, particularly in emotional well-being, interpersonal relationships, and autonomy.^8^ Despite its relevance, MHL is not adequately assessed among pwMS, resulting in a knowledge gap among health care professionals (HCPs) involved in their care. Among HCPs, MS nurse specialists play a pivotal role in promoting MHL and addressing unmet psychological needs.^9,10^ As frontline professionals with continuous patient contact, nurses are uniquely positioned to identify early signs of emotional distress, normalize conversations about MH, and support patients in learning skills to manage MS-related psychological distress while adopting a multidisciplinary care approach.^11,12^ Nevertheless, research on MHL in MS and the role of the nurses remains limited. Only one recent study has directly examined MHL in pwMS, reporting a significant association between MHL and health-promoting behaviors in this population^7^; however, no studies to date have evaluated MHL in relation to the role of nurses. Additional research has focused on health literacy,^13,14^ which refers to an individual’s ability to access, understand, appraise, and use health information to make informed decisions about their care.^15^ Among MHL, the role of nurses has been studied primarily in other populations, such as students and HCPs.^16,17^ By stratifying pwMS according to their MHL profiles, nurses can tailor educational interventions, strengthen help-seeking behaviors, and enhance engagement in care pathways. Therefore, this study aims to investigate MHL levels in pwMS and their possible correlation with QoL, sociodemographic factors, and the role of MS nurses in supporting MHL development.

## Methods

A multicenter, observational, cross-sectional descriptive online survey was conducted between March and August 2024. The STrengthening the Reporting of Observational Studies in Epidemiology (STROBE) guidelines were used for reporting.^18^ The study was conducted through the Italian Network of Multiple Sclerosis Nurses, involving multiple health care facilities located in 18 regions of northern, central, and southern Italy. A convenience sampling technique was employed. Adult pwMS were invited to participate and were included if they had a confirmed MS diagnosis according to the 2017 McDonald criteria, were at least 18 years old, provided informed consent, and had no severe cognitive impairment that could interfere with completing the questionnaire. Patients who did not meet these criteria were excluded. Trained research nurses were responsible for recruiting participants individually and verifying that the inclusion criteria were met. Participants who agreed to take part in the study were provided with detailed study information and informed that their participation was voluntary and anonymous.

Data were collected on sociodemographic and clinical characteristics, as well as patients’ experience with nursing care. MHL was assessed using the Mental Health Literacy Questionnaire-Short Version for adults (MHLq-SVa),^19^ a 16-item instrument measuring 4 dimensions: knowledge of MH problems, erroneous beliefs and stereotypes, help-seeking and first aid skills, and self-help strategies. Each item is rated on a 5-point Likert scale. QoL was measured with the Multiple Sclerosis Quality of Life Questionnaire (MS-QLQ27),^20^ a 27-item instrument assessing the impact of MS on physical, psychological, social, and treatment-related domains. Items are rated on a 5-point scale, and a global score was calculated as the mean of all valid items. In this study, the Italian versions of both instruments underwent forward-backward translation, expert review for content and face validity, and pilot testing to ensure semantic and cultural adequacy. The MHLq-SVa was selected because it is a brief, multidimensional tool specifically developed to measure core components of MHL, including knowledge, stereotypes, help-seeking, and self-help. The original validation demonstrated strong psychometric performance (CFI=0.95; NNFI=0.94-0.96; RMSEA=0.04-0.05) and high factor loadings (0.50-0.97); Cronbach’s α ranged from 0.59 to 0.93 across subscales. In this study, internal consistency was good (α=0.816), with EFA supporting factorability (Bartlett *P*<0.001; KMO=0.779) and CFA showing acceptable model fit (CFI=0.905; RMSEA=0.0724). The MS-QLQ27 was chosen because it is an MS-specific, brief, and comprehensive instrument capturing physical, psychological, social, and work-related aspects of QoL. Prior validation showed excellent reliability (α=0.93-0.94) and strong convergent and known-groups validity. In this study, internal consistency was excellent (α=0.939), EFA supported suitability for factor analysis (Bartlett *P*<0.001; KMO=0.896), and, as expected based on the original development, the CFA did not support a stable multidimensional structure, confirming the appropriateness of using the global score. For the sample size calculation, a priori power analysis was conducted using G*Power 3.1, adopting a medium effect size (Cohen’s *f*=0.25), α=0.05, and 80% statistical power. The analysis was based on an ANOVA framework, reflecting the aim of this study: to compare MHLq-SVa scores across 3 education levels, a sociodemographic factor consistently associated with MHL in the literature. Under these parameters, the minimum required sample size was 159 participants. In addition, for the psychometric validation of the MHLq-SVa in pwMS, methodological guidelines recommend recruiting 5 to 10 participants per item.^21^ Given the 16 items of the scale, this corresponds to a minimum of 160 participants.

Descriptive statistics were used to summarize the data. The Mann-Whitney *U* test assessed differences between groups for non-normally distributed continuous variables, and Spearman’s rho to evaluate associations between continuous or ordinal variables. All statistical analyses were performed using IBM SPSS Statistics, version 28.0. A 2-tailed *P* value of <0.05 was considered statistically significant. The study was approved by the Italian Multiple Sclerosis Nurses Society and conducted in accordance with national and European Union regulations.

## Results

A total of 170 patients completed the questionnaire (34% response rate). Among these 151 (88.8%) were females with a mean age of 36.5 (11.2) years. Concerning educational attainment, 61 (35.9%) had a bachelor’s degree, and 156 (91.8%) were diagnosed with relapsing-remitting MS (Table 1). In terms of patient experience with nursing care, 152 (89.4%) found the information about the psychological and mental aspect provided by nurses to be clear, and 156 (91.8%) reported that nurses were available to answer their questions (Supplemental Table 1, Supplemental Digital Content 1, http://links.lww.com/JNN/A644). The mean MHLq-SVa total score was 67.1 (6.4). The MS-QLQ27 score had a mean of 2.3 (0.7) (Table 2). Females reported higher MHLq-SVa total score (*U*=1029.0, *P*=0.045), greater knowledge of MH problems (*U*=971.5, *P*=0.021), and fewer erroneous beliefs/stereotypes (*U*=971.0, *P*=0.019) compared with males. Participants with higher educational levels showed significantly better scores in knowledge of MH problems (*H*=9.032, *P*=0.029), fewer erroneous beliefs/stereotypes (*H*=15.469, *P*=0.001), and greater self-help strategies (*H*=12.950, *P*=0.005). The total MHLq-SVa score (*H*=17.685, *P*=0.001) and the MS-QLQ27 score (*H*=13.860, *P*=0.003) also differed significantly across education levels (Supplemental Table 2, Supplemental Digital Content 2, http://links.lww.com/JNN/A645). The total MHLq-SVa score was strongly correlated with all its dimensions: knowledge of MH problems (ρ=0.797), erroneous beliefs/stereotypes (ρ=0.708), help-seeking and first aid skills (ρ=0.514), and self-help strategies (ρ=0.681), all *P*<0.001. MS-QLQ27 showed a weak correlation only with knowledge of MH problems (ρ=0.156, *P*=0.042), with no significant associations with other MHL dimensions or with the total MHLq-SVa score (Supplemental Figure 1, Supplemental Digital Content 3, http://links.lww.com/JNN/A646). Mann-Whitney *U* tests showed no significant differences in any MHLq-Sva subscale or in the total score across all indicators of nursing care (all *P*>0.05). The MS-QLQ27 global score was also not associated with nursing care evaluations, except for a single significant difference related to nurses’ availability, with lower scores among patients who perceived nurses as less available (*U*=678.5, *P*=0.048) (Supplemental Table 3, Supplemental Digital Content 4, http://links.lww.com/JNN/A647).

**TABLE 1:** Sociodemographic and Clinical Characteristics of Patients With Multiple Sclerosis (n=170)

Characteristics	n (%)
Mean age (SD)	36.5 (11.2) years
Sex	
Female	151 (88.8)
Male	19 (11.2)
Educational level	
Lower secondary education	16 (9.4)
Upper secondary school diploma	81 (47.6)
Bachelor’s degree	61 (35.9)
Master’s degree/PhD	12 (7.1)
Employment status	
Employed	118 (69.4)
Unemployed	52 (30.6)
Marital status	
Unmarried	89 (52.4)
Married/cohabiting	72 (42.4)
Separated/divorced	8 (4.7)
Widow/widower	1 (0.6)
Mean age at MS onset (SD)	28.3 (8.6) years
Type of MS	
CIS/RIS	1 (0.6)
RRMS	156 (91.8)
PPMS	5 (2.9)
SPMS	8 (4.7)
Disease-modifying treatment	
Anti-CD52 monoclonal antibody	3 (1.8)
Immune reconstitution therapy (IRT)	22 (12.9)
Dimethyl fumarate	33 (19.4)
Glatiramer acetate	3 (1.8)
Interferon	3 (1.8)
Natalizumab	33 (19.4)
No treatment	11 (6.5)
Anti-CD20 monoclonal antibodies	37 (21.7)
S1P receptor modulators	15 (8.8)
Teriflunomide	10 (5.9)
Geographical area	
Northern Italy	86 (50.6)
Central Italy	33 (19.4)
Southern Italy	51 (30.0)
Presence of specialized nurses in the MS center	
Yes	146 (85.9)
No	24 (14.1)

CIS = Clinically Isolated Syndrome; MS = multiple sclerosis; PPMS = primary progressive multiple sclerosis; RIS = radiologically isolated syndrome; RRMS = relapsing-remitting multiple sclerosis; SPMS = secondary progressive multiple sclerosis.

**TABLE 2:** Descriptive Statistics of Mental Health Literacy (MHLq-SVa) Dimensions and MS-QLQ27 (n=170)

Instruments/Domains	Mean (SD)
Knowledge of mental health problems	24.3 (3.1)
Erroneous beliefs/stereotypes	12.9 (2)
Help-seeking and first aid skills	12.8 (2)
Self-help strategies	17.1 (2.2)
Total MHLq-SVa score	67.1 (6.4)
MS-QLQ27 score	2.3 (0.7)

MHLq-SVa = Mental Health Literacy Questionnaire-Short Version for adults; MS-QLQ27 = Multiple Sclerosis Quality of Life Questionnaire.

## Discussion

This study investigated MHL levels in pwMS using the MHLq-SVa and examined their association with QoL and sociodemographic factors. Assessing patient experience with nursing care provides important contextual information. A generally high satisfaction level with nursing interventions was observed, particularly regarding the clarity of information provided, the nurse’s availability to address questions, and the courteousness and respectfulness of interactions. These findings are important, as effective communication and supportive relationships are key to patient education and empowerment, both closely tied to MHL development.^8^ Recent evidence shows that MH nurses shape patients’ MH understanding and integrate sociocultural factors into health literacy practices.^22^ Therefore, the positive patient experiences observed in this study may reflect not only the technical competence of nursing care but also its potential role in enhancing MH awareness and early help-seeking behaviors, which are crucial components of improved MH outcomes.

The assessment of MHL among pwMS revealed moderate overall levels, with notable variation across domains. Participants showed relatively good knowledge of MH problems but lower scores in misconceptions, stereotypes, and help-seeking attitudes. These findings align with prior research suggesting that, despite increased awareness, stigma and misinformation still hinder symptom recognition and timely care.^23^ Similar trends have been observed in pwMS, where partial knowledge coexists with persistent misunderstandings and limited appreciation for psychological support.^24^ These gaps in MHL highlight an important opportunity for MS nurses to provide corrective education, address stigma, and promote evidence-based help-seeking behaviors within routine MS care. Such misconceptions may exacerbate distress and delay intervention, especially in those with chronic neurological conditions. QoL among participants varied considerably, reflecting MS’s broad impact on functioning, emotional well-being, and social life, consistent with existing literature linking disability, psychological distress, and social isolation to lower QoL.^20^ Recent studies have emphasized the bidirectional relationship between MHL and QoL: higher MHL is associated with more adaptive coping strategies, greater psychological resilience, and improved subjective well-being.^25,26^ Nurses can play a pivotal role in reinforcing these mechanisms by integrating supportive counseling and self-management training into standard MS care pathways. The analysis showed that female patients had significantly higher MHL than males, particularly in terms of greater MH knowledge and fewer stereotypical beliefs, consistent with prior studies reporting higher MHL among women.^27^ Higher MHL in women may act as a protective factor, and these findings support earlier research indicating that men with low MHL are at greater risk of unmet mental health care needs.^28^ Targeted, nurse-led educational interventions may therefore be essential to reduce gender disparities in MHL and improve engagement in MH care among male patients. Among pwMS, who face both physical and psychological challenges, low MHL in men may further limit access to MH support. Enhancing MHL among male patients could reduce gender disparities in MH outcomes and improve MS management.

Educational level significantly influences both MHL and perceived QoL. Participants with higher education exhibited greater knowledge of MH problems, fewer misconceptions and stereotypes, and better self-help abilities, according to previous research.^29^ However, no differences emerged across education levels in help-seeking and first aid skills, suggesting that these may rely more on psychosocial factors like past health care experiences or social support than on formal education. Furthermore, participants with higher education levels reported significantly greater self-help competence, reinforcing the idea that education empowers individuals with SM to adopt more proactive strategies in managing their MH alongside their physical condition.^30^ These findings underline the key role of nurse-delivered education in supporting people with lower education and improving equal access to MH knowledge. The overall MHLq-SVa score confirmed that educational attainment is a strong predictor of general MHL competence. Regarding QoL, those with lower education reported poorer outcomes, reflecting evidence that lower socioeconomic status, often linked to education, is associated with worse MH and well-being.^31^ These findings underscore the need to address educational disparities when developing MH promotion strategies for pwMS. MS nurses, who routinely provide personalized education, are ideally placed to tailor interventions based on patients’ educational backgrounds and learning needs. The MHLq-SVa total score showed strong, significant positive correlations with all 4 dimensions, supporting the view of MHL as an integrated, multidimensional construct. The instrument also demonstrated excellent psychometric properties, confirming its reliability. However, the association between MHL and QoL was limited. Only the “knowledge of mental health problems” dimension showed a weak but significant positive correlation with QoL, suggesting that better MH knowledge may slightly enhance perceived well-being. No significant correlations emerged between QoL and the other MHL dimensions, indicating that factors such as accurate beliefs or help-seeking skills may not directly translate into better well-being, possibly due to barriers such as limited service access, disability, or a lack of support.^32^ These findings suggest that nurses should not assume that improving MHL alone will enhance QoL; instead, nursing interventions should integrate psychological support, symptom management, and care coordination to maximize patient well-being. Overall, MHL did not show a direct impact on QoL. It is likely that QoL in MS is more strongly influenced by disease-specific factors (eg, physical impairment and fatigue) or psychological dimensions (eg, coping and self-efficacy), which were not assessed in this study. This highlights an avenue for advanced nursing practice, where MS nurses can integrate MHL assessment with broader biopsychosocial evaluations to better address the complex determinants of QoL among pwMS. Finally, the absence of significant associations between patients’ evaluations of nursing care and MHLq-SVa scores may indicate that MS nurses are not yet fully equipped to recognize or influence patients’ MHL. This could reflect a broader gap in training, suggesting the need for targeted educational interventions to strengthen nurses’ competencies in assessing and supporting MHL in clinical settings. Interestingly, the only significant finding emerged for QoL; patients who perceived nurses as less available reported higher mean ranks in the MS-QLQ27 score. Although counterintuitive, this may reflect clinical or emotional complexities, such as patients with poorer well-being requiring more support and therefore perceiving staff as less available. Given that patients and nurses often hold different perceptions of care quality due to their distinct backgrounds and experiences,^33^ this finding underscores the need to further explore how nursing workload, communication, and patient expectations interact to shape perceived support and outcomes among MHL.

This study offered a comprehensive assessment of MHL and QoL in pwMS. However, several limitations should be acknowledged. The convenience sampling employed in this study is susceptible to bias and lacks the generalizability of random sampling, as it does not accurately represent the broader population. The cross-sectional design restricts the ability to infer causality between MHL and QoL. Self-reported measures may be subject to recall or social desirability bias, which can affect response accuracy. A further limitation is that only age, educational level, and employment status were analyzed as sociodemographic variables. These were the only characteristics consistently collected at all participating centers; however, this narrow focus limited the ability to consider other potentially relevant sociodemographic factors that could affect MHL in pwMS. Finally, the modest correlations between MHL dimensions and QoL suggest that other clinical or psychosocial variables (eg, depression and anxiety), not assessed in this study, may also play a significant role in patients’ QoL and serve as potential confounders.

## Conclusions

This study underscores the impact of sociodemographic factors such as sex and education on MHL and QoL in pwMS. Higher MHL levels correlated with better knowledge, fewer misconceptions, and stronger self-help skills, though not always with improved perceived QoL. These findings indicate that while enhancing MHL is essential for empowering patients in managing their MH, other factors must also be addressed to improve overall well-being. Nurses play a central role in this process and are well-positioned to assess MHL. Assessing MHL can help nurses identify MS patients who may require targeted education to recognize psychological symptoms earlier. Enhancing MHL through nurse-led interventions may promote timely help-seeking and more effective self-management. Therefore, incorporating structured MHL support into standard MS care can improve psychological well-being and overall QoL.

The validated MHLq-SVa supports early identification of patients with low MHL, enabling tailored interventions. Strengthening the role of nurses within multidisciplinary teams and equipping them with tools such as the MHLq-SVa represent key strategies to bridge gaps in MHL and enhance QoL in pwMS. Future research should integrate clinical severity measures and psychosocial contextual factors to provide a more comprehensive understanding of MHL in MS nursing clinical practice.

## Supplementary Material
