# Supplementary material for: Exploring Mental Health Literacy and Quality of Life in Multiple Sclerosis: A Cross-Sectional Study
Source: J Neurosci Nurs. 2026 Feb 25;58(3):118–23. doi: 10.1097/JNN.0000000000000880 (PMC13132064; doi:10.1097/JNN.0000000000000880)
Supplement: Supplementary file 1 [file jnn-58-118-s001.docx]

**Supplemental Digital Content 1.**

Supplemental Table 1. Patients’ evaluation of nursing communication, availability, courtesy, and overall experience (n=170).

| **Variables** | **n (%)** |
| --- | --- |
| **Were the information provided by nurses clear?** |  |
| Yes | 152 (89.4) |
| No | 18 (10.6) |
| **Were the nurses available to answer your questions about MS management?** |  |
| Yes | 156 (91.8) |
| No | 14 (8.2) |
| **Were the nurses courteous and respectful?** |  |
| Yes | 161 (94.7) |
| No | 9 (5.3) |
| **Overall, would you consider your experience to be positive?** |  |
| Yes | 158 (92.9) |
| No | 12 (7.1) |
