# Supplementary material for: Exploring Mental Health Literacy and Quality of Life in Multiple Sclerosis: A Cross-Sectional Study
Source: J Neurosci Nurs. 2026 Feb 25;58(3):118–23. doi: 10.1097/JNN.0000000000000880 (PMC13132064; doi:10.1097/JNN.0000000000000880)
Supplement: Supplementary file 2 [file jnn-58-118-s002.docx]

**Supplemental Digital Content 2.**

| Supplemental Table 2. Non-parametric comparisons (Mann-Whitney and Kruskal-Wallis tests) and significance levels across sociodemographic variables (n=170). | | | | | | | | | | | |
| --- | --- | --- | --- | --- | --- | --- | --- | --- | --- | --- | --- |
| **Variables/**  **Instruments/**  **Dimensions** | **Gender** | | | **Employment status** | | | **Educational Level** | | | | |
|  | **Mean Rank** | | **P** | **Mean Rank** | | **P** | **Mean Rank** | | | | **P** |
|  | **F** | **M** |  | **E** | **U** |  | **LSE** | **USSD** | **BD** | **MDP** |  |
|  | (n=151) | (n=19) |  | (n=118) | (n=52) |  | (n=16) | (n=81) | (n=61) | (n=12) |  |
| **Knowledge of mental health problems** | 88.57 | 61.13 | .021* | 81.95 | 93.56 | .154 | 66.56 | 87.14 | 81.42 | 120.46 | .029* |
| **Erroneous beliefs/**  **stereotypes** | 88.57 | 61.11 | .019* | 83.04 | 91.09 | .313 | 55.47 | 86.84 | 83.42 | 127.08 | .001* |
| **Help-seeking and first aid skills** | 86.65 | 76.39 | .381 | 85.82 | 84.78 | .897 | 76.72 | 88.80 | 78.62 | 109.92 | .153 |
| **Self-help strategies** | 83.77 | 99.26 | .185 | 84.31 | 88.21 | .625 | 56.00 | 91.57 | 79.31 | 115.33 | .005* |
| **Total**  **MHLq-SVa Score** | 88.19 | 64.16 | .045* | 82.49 | 92.33 | .229 | 59.03 | 89.73 | 77.61 | 132.29 | .001* |
| **MS-QLQ27 Score** | 83.26 | 103.26 | .095 | 80.42 | 97.04 | .042 | 107.38 | 94.31 | 67.60 | 87.88 | .003* |
| F: Female; M: Male; E: Employed; U: Unemployed; LSE: Lower secondary education; USSD: Upper secondary school diploma; BD: Bachelor's degree; MDP: Master's degree/PhD; * p < 0.05, significance level (two-tailed test). | | | | | | | | | | | |
