# Supplementary material for: Exploring Mental Health Literacy and Quality of Life in Multiple Sclerosis: A Cross-Sectional Study
Source: J Neurosci Nurs. 2026 Feb 25;58(3):118–23. doi: 10.1097/JNN.0000000000000880 (PMC13132064; doi:10.1097/JNN.0000000000000880)
Supplement: Supplementary file 3 [file jnn-58-118-s003.docx]

**Supplemental Digital Content 3.**

**
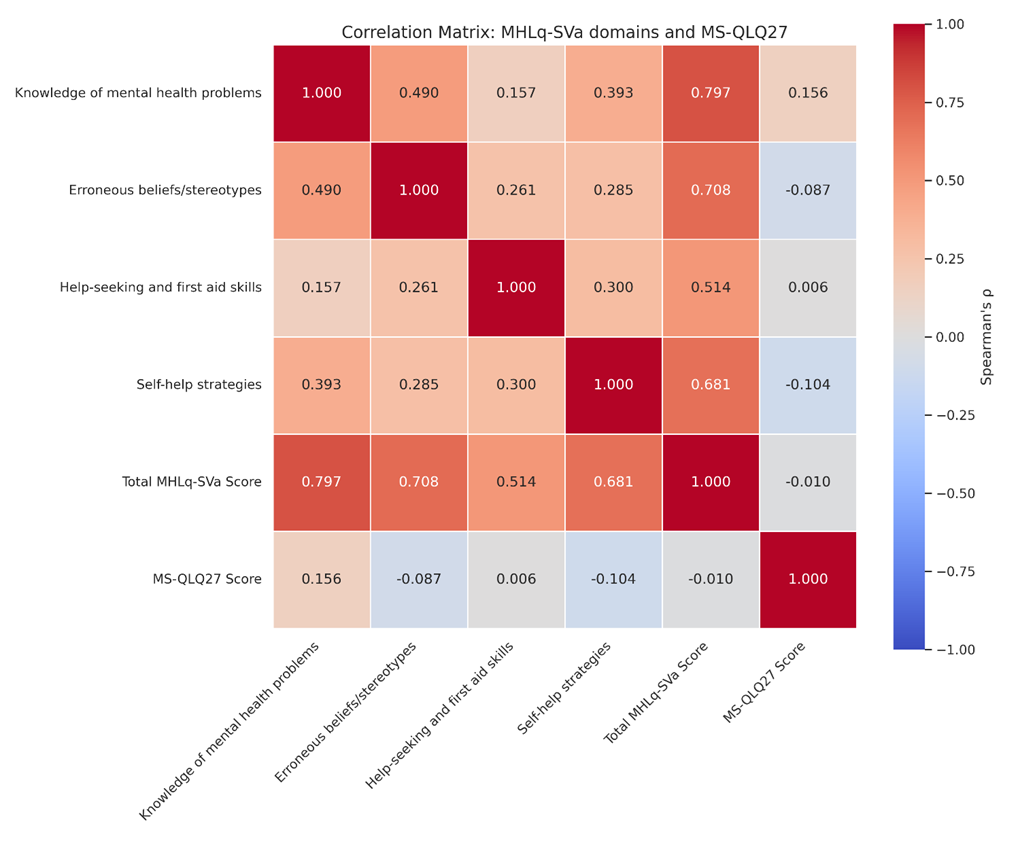
**

Supplemental Figure 1. Correlation Matrix between MHLq-SVa domains and MS-QLQ27.
