# Supplementary material for: Exploring Mental Health Literacy and Quality of Life in Multiple Sclerosis: A Cross-Sectional Study
Source: J Neurosci Nurs. 2026 Feb 25;58(3):118–23. doi: 10.1097/JNN.0000000000000880 (PMC13132064; doi:10.1097/JNN.0000000000000880)
Supplement: Supplementary file 4 [file jnn-58-118-s004.docx]

**Supplemental Digital Content 4.**

| Supplemental Table 4. Non-parametric comparisons between nursing care evaluation indicators and outcome measures (MHLq-Sva and MS-QLQ27) (n=170). | | | | | | | | | | | | |
| --- | --- | --- | --- | --- | --- | --- | --- | --- | --- | --- | --- | --- |
| **Variables/**  **Instruments/**  **Dimensions** | **Clarity of information** | | | **Nurses’ availability** | | | **Courtesy and respect** | | | **Overall positive experience** | | |
|  | **Mean Rank** | | **P** | **Mean Rank** | | **P** | **Mean Rank** | | **P** | **Mean Rank** | | **P** |
|  | **YES** | **NO** |  | **YES** | **NO** |  | **YES** | **NO** |  | **YES** | **NO** |  |
|  | (n=152) | (n=18) |  | (n=156) | (n=14) |  | (n=161) | (n=9) |  | (n=158) | (n=12) |  |
| **Knowledge of mental health problems** | 86.43 | 77.67 | .473 | 84.79 | 93.39 | .529 | 85.08 | 93.06 | .634 | 84.32 | 101.00 | .255 |
| **Erroneous beliefs/**  **stereotypes** | 85.70 | 83.83 | .876 | 85.77 | 82.46 | .805 | 85.64 | 83.06 | .875 | 84.34 | 100.75 | .253 |
| **Help-seeking and first aid skills** | 85.43 | 86.11 | .954 | 84.15 | 100.57 | .220 | 84.82 | 97.72 | .433 | 85.56 | 84.75 | .955 |
| **Self-help strategies** | 87.78 | 66.22 | .072 | 85.56 | 84.86 | .958 | 84.98 | 94.89 | .547 | 84.13 | 103.54 | .177 |
| **Total**  **MHLq-SVa Score** | 86.53 | 76.83 | .429 | 84.67 | 94.75 | .462 | 85.24 | 90.17 | .770 | 84.15 | 103.33 | .192 |
| **MS-QLQ27 Score** | 82.80 | 104.71 | .080 | 82.85 | 110.81 | .048* | 83.73 | 107.50 | .156 | 83.40 | 108.00 | .107 |
| * p < 0.05. significance level (two-tailed test). | | | | | | | | | | | | |
